# Modified e-Delphi Process for the Selection of Patient-Reported Outcome Measures for Children and Families With Type 1 Diabetes Using Continuous Glucose Monitors: Delphi Study

**DOI:** 10.2196/38660

**Published:** 2022-11-30

**Authors:** Payal Shah, Jennifer K Raymond, Juan Espinoza

**Affiliations:** 1 Children's Hospital Los Angeles Los Angeles, CA United States

**Keywords:** type 1 diabetes, diabetes, diabetic, juvenile, pediatrics, paediatrics, child, youth, continuous glucose monitor, glucose, monitoring, patient reported, outcome measure, PROM, Delphi, disease management, self-management, measurement, instrument

## Abstract

**Background:**

Type 1 diabetes (T1D) management is complex and associated with significant psychosocial burden. Continuous glucose monitors (CGM) can improve disease management and outcomes and introduce new or exacerbate existing psychosocial concerns. Patient-reported outcome measures (PROMs) can be used to capture this information, but there is no consensus on which PROMs should be used in pediatric CGM research.

**Objective:**

Here we describe the process to (1) identify PROMs that could be used to assess the impact of CGMs on pediatric patients with T1D, (2) implement a modified electronic Delphi (e-Delphi) methodology to arrive at an expert consensus on which PROMs are most suitable for clinical and research applications, and (3) establish a periodicity table for the administration of PROMs over time in a real-world evidence study.

**Methods:**

To identify appropriate PROMs for pediatric patients and families with T1D and CGMs, we conducted an asynchronous, e-Delphi process with a multidisciplinary group of experts from around the country. We identified candidate instruments through a literature review. The 3-round e-Delphi process was conducted via a study website, email, and web-based forms. Participants provided opinions on the usefulness of instruments, age validation, feasibility, time, and frequency of administration.

**Results:**

In total, 16 experts participated in the e-Delphi process; 4 of whom consistently participated in all 3 rounds. We identified 62 candidate instruments, which were narrowed down to 12 final PROMs across 5 domains: diabetes distress and burden (n=4), autonomy (n=2), quality of life (n=1), psychosocial (n=3), and technology acceptance (n=2). A quarterly administration schedule was developed to reduce burden on participants.

**Conclusions:**

PROMs can provide critical insights into the psychosocial well-being of patients. The specific measures identified in the paper are particularly well suited for pediatric patients with T1D using CGMs. Clinical implementation could help health care providers, patients, and families to engage in more comprehensive disease management.

## Introduction

Type 1 diabetes (T1D) impacts nearly 1.6 million Americans, including approximately 187,000 children and adolescents [1]. The advancement of diabetes technology, specifically continuous glucose monitors (CGM), has made self-management and home monitoring more feasible and accessible [2,3]. Disease management can be complex and includes the management of blood sugar levels with insulin, diet plans, exercise, and a coping lifestyle to prevent complications [1]. This complexity can lead to distress, depression, anxiety, eating disorders, poor treatment satisfaction, and adherence in patients and their families [1,4]. Therefore, chronic disease management requires an integrated approach with routine management as well as proactive risk assessment. Patient-reported outcome measures (PROMs) can facilitate systematic assessment of patients’ and parents’ perception of a child's overall well-being and deeper understanding of the patient experience. The Center for Devices and Radiological Health, part of the US Food and Drug Administration (FDA), has emphasized the use of PROMs in medical device evaluation and regulatory decisions to support claims in approved medical product labeling in pre- and postmarket medical device–related clinical studies [4-8].

There have been several PROMs developed for patients with diabetes, though they are rarely implemented outside of research settings [9]. The Department of Public Health of the University of Oxford has published a list of recommended PROMs for use in the management of diabetes after structured systematic review in 2006 and 2009 [10]. The FDA has qualified the Insulin Dosing Systems: Perceptions, Ideas, Reflections, and Expectations (INSPIRE) questionnaires as a medical device development tool to assess the impact of automated insulin dosing (AID) systems on psychosocial functioning and quality of life (QoL) [11]. For pediatric patients, it is not as simple as reusing adult instruments. Pediatric PROMs need to be designed to capture a parent or caretaker’s perspective, consider parent-child dynamics, and accommodate a broad neurodevelopmental spectrum and varying age-appropriate literacy and numeracy skills [12,13]. These psychometric challenges, along with overall less funding and focus on pediatric research compared to adults [14-16], have contributed to a lag in the development and adoption of pediatric PROMs.

Since 2018, we have been working on an FDA-funded real-world evidence study focused on children with T1D using CGMs, with the ultimate goal of creating a real-time, prospective database of patients using medical devices that can be used for clinical, operational, research, and regulatory purposes. One key component of the project is to aggregate data from several sources, including the electronic health record (EHR), medical devices, and the patients themselves. To that end, we have leveraged a number of technologies, including Cerner’s population health management platform, HealtheIntent, to ingest clinical data, integration engines to ingest CGM data directly into the EHR [17], and REDCap to collect patient-reported outcomes [18,19]. Here we describe out process to (1) identify PROMs that could be used to assess the impact of CGMs on pediatric patients with T1D, (2) implement a modified electronic Delphi (e-Delphi) methodology to arrive at an expert consensus on which PROMs are most suitable for clinical and research applications, and (3) establish a periodicity table for the administration of PROMs over time in our real-world evidence study. The Delphi technique, developed in the 1950s, is a structured process that leverages the judgment of experts through a series of rounds that integrate controlled feedback to develop consensus on a specific topic [20-22]. The overarching goal of this Delphi process is the creation of a battery of PROMs to facilitate patient-provider interaction and individualized patient-centered care, which can reflect measurable changes in population health over time.

## Methods

### PROMs Identification and Literature Review

We collaborated with a medical librarian to systematically search PubMed (National Library of Medicine), Embase (Elsevier), Web of Science (Clarivate Analytics), Engineering Village (Elsevier), and ClinicalTrials.gov (National Library of Medicine) to identify relevant publications related to PROMs, CGMs, and pediatrics (referring to individuals aged 0-18 years). We ran a series of searches using a combination of controlled vocabulary (when available) and keywords to capture multiple facets of PROMs which included the following: PROMs, questionnaires, pain, sleep and fatigue, stigma, self-efficacy and relationships, physical activity, stress, cognition, and emotions. The queries were not limited by publication date.

Because this study focused on CGMs and PROMs, studies focused on insulin pumps, AIDs, and other diabetes technologies were excluded. Search results underwent title and abstract screening for relevance, and full-text review was conducted by 2 independent researchers to identify relevant PROMs. This process was facilitated by the medical librarian, and a third researcher was available to adjudicate as needed. Citations in included papers were also reviewed for eligibility. Exclusion criteria were as follows: duplicate instruments, inability to find the full text of the instruments, instruments that were not validated, older versions of PROMs, and instruments that were too long to administer in a clinical setting. All PROMs were grouped by the study team into one of 5 domains: autonomy, psychosocial factors, diabetes distress and burden, general health and QoL, and technology and acceptance.

### Delphi Expert Panel Recruitment

Potential participants were identified by the study team using purposive sampling without quotas. Participants were eligible if they were directly involved in the care or support of pediatric patients with T1D using CGMs, including experienced clinicians, researchers, psychologists, health educators, and device developers. Participants were recruited by email invitation describing the aims of the study, purpose of the PROMs, study design, participation details, and anticipated time commitment. Snowball recruitment was used to identify additional participants beyond the first round of invitations.

### e-Delphi Process

**Figure 1:**
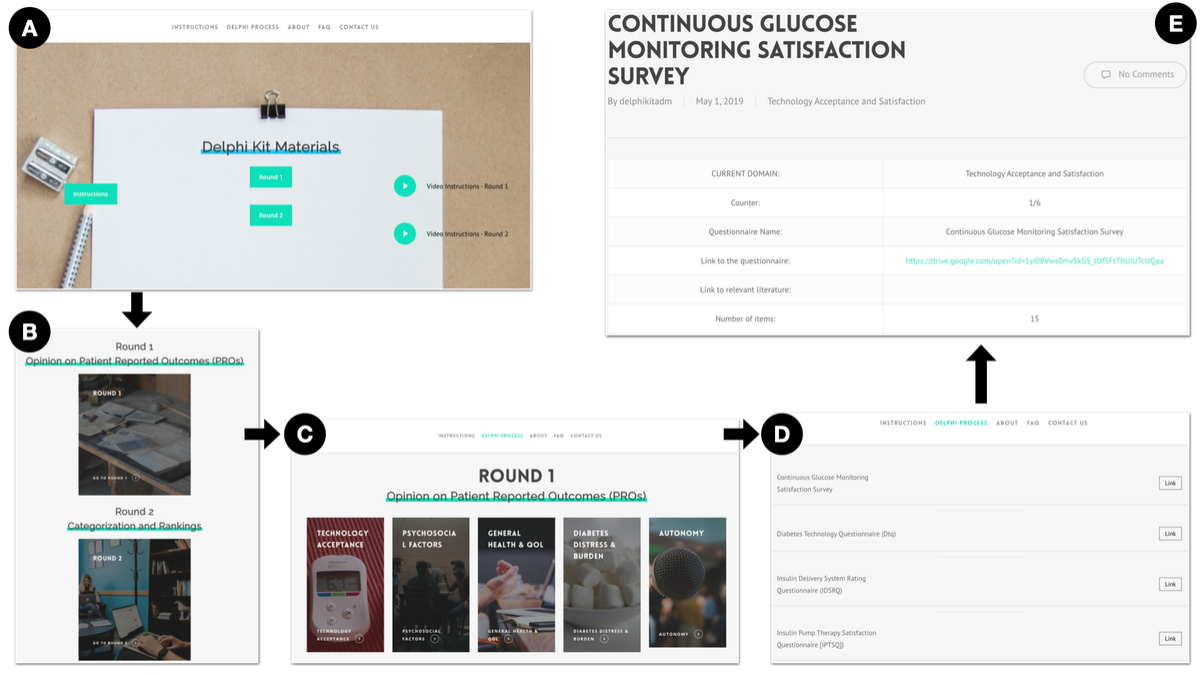
Overview of the Delphi Kit website [23] used for organizing the Delphi process and collecting data. The website included multiple pages encompassing information on the Delphi process, description of the study, instructions for participants, survey pages, and the first 2 rounds of the electronic Delphi process. (A) The home page, (B) the Delphi process page, (C) the round 1 page, (D) the technology acceptance domain page, and (E) the Continuous Glucose Monitoring Satisfaction Survey page.

**Figure 2:**
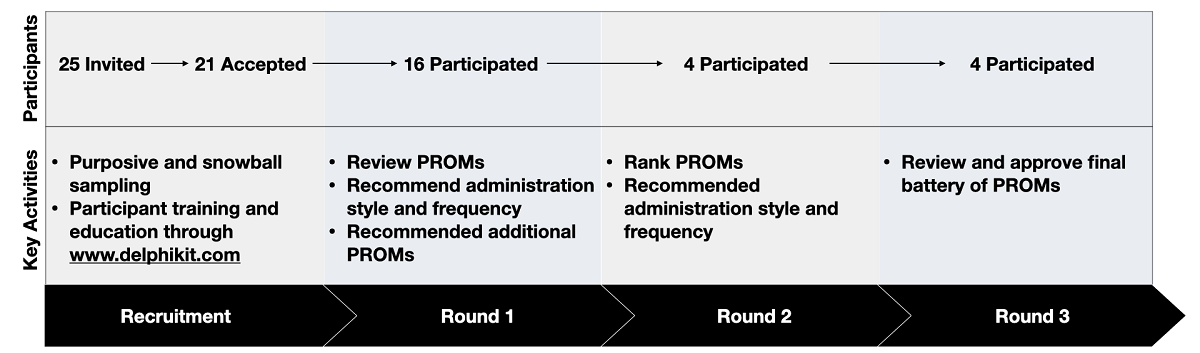
Key activities and engagement of participants across each stage of the electronic Delphi process. PROM: patient-reported outcome measure.

All parts of the e-Delphi process were conducted asynchronously and on the internet. The website and web-based data collection tools are hosted on the Delphi Kit website (Figure 1) [23]. Delphi panelists were sent instructions and a video explaining how to use the website. The e-Delphi process consisted of 3 rounds of feedback that needed to be provided on the internet (Figure 2). During each round, participants were given a 4-week period and reminders to nonresponders were sent weekly. Participants who failed to respond despite 3 email reminders were defined as withdrawals. Participants could also withdraw on their own request if they could not commit the time. Data collected up to that point was included for analysis. Demographics were collected from all Delphi participants.

In the first round, the Delphi website included detailed information about each instrument with a description, number of items, link to the full text questionnaire, and references. Participants were shown all included PROMs and were asked to provide their opinion on each instrument, and if they had any experiences administering it in clinical or research settings. They were also asked if the instrument should be administered at a specific time or milestone during the study, or if it should be administered like an ecological momentary assessment—a brief, repeated instrument, typically triggered by a specific event, completed by the subject in their natural environment [24]. Finally, participants were asked to suggest other instruments not included in round 1. The research team reviewed and categorized the responses from round 1 and refined the list of PROMs for round 2. In round 2, participants were asked to rank the remaining instruments within each domain on the basis of multiple factors, including robustness of the research data obtained using the instrument, the importance or relevance of the concept or phenomenon being addressed, feasibility of administering the instrument, and overall burden on patients and providers. They were also asked to suggest how often each instrument should be administered or if they should be administered after specific clinical events (hospitalization, emergency room visit, new prescription, etc). An open-ended question for general feedback was included as well. We reviewed all responses and further refined the list of PROMs for round 3. In round 3, the final list of PROMs along with their proposed administration frequency and timeline was sent to all reviewers for feedback. Instruments were evaluated on the basis of multiple factors, including the number of items, ease and time of completion, age range, parent versus self-report, and availability (ie, cost of proprietary instruments). The participants and the responses were anonymized to each other.

### Ethical Considerations

This study is not considered human subjects research; therefore, no consent was obtained and no ethics approval was required.

## Results

### e-Delphi Process and Results

A total of 25 participants were invited to participate in the Delphi process, of whom 21 agreed to participate (Figure 2). There was participant attrition after each round; only 4 participants completed the third and final rounds. The core research team (JE, JR, and PS) reviewed and integrated all responses after each round.

### e-Delphi Process

**Figure 3:**
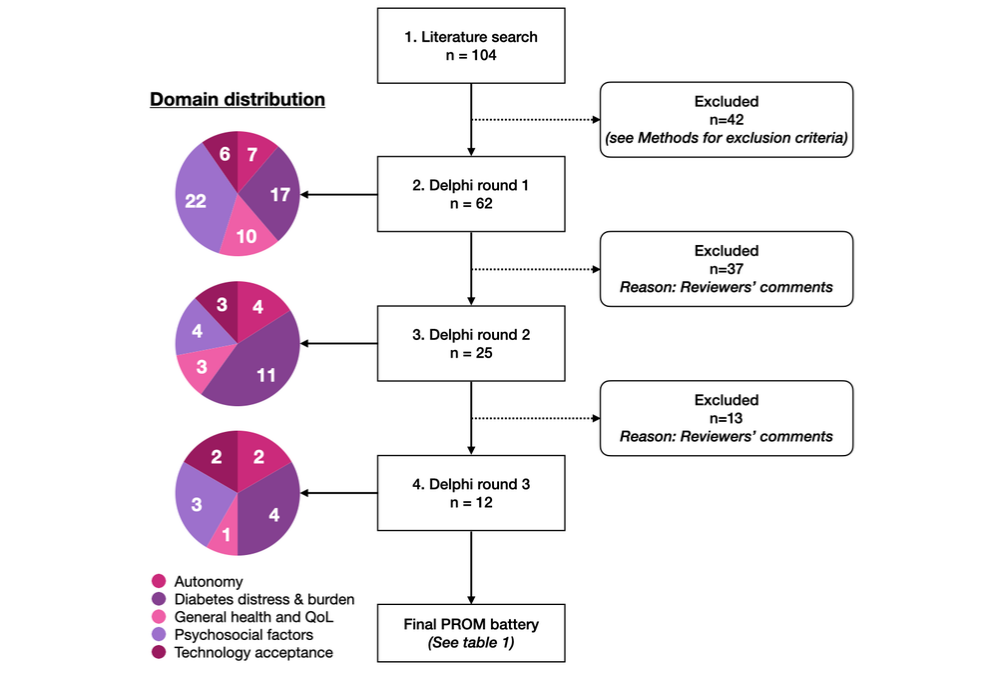
Modified Preferred Reporting Items for Systematic Reviews and Meta-Analyses diagram describing the different rounds of the electronic Delphi process and the number of patient-reported outcome measures selected by domain distribution: autonomy, diabetes distress and burden, general health and quality of life, psychosocial factors, and technology acceptance. PROM: patient-reported outcome measure; QoL: quality of life.

Our literature review identified a total of 104 relevant articles. After applying all of the exclusion criteria, 62 unique PROMs were included in round 1. Figure 3 shows the flow of PROMs across rounds and how they are distributed across domains. In round 1, all participants reviewed the instruments and references. In total, 37 PROMs were excluded on the basis of feedback, primarily owing to lack of fit, unfamiliarity, being too lengthy, or being too difficult to administer in a real-world setting. The core research team reviewed all the feedback and identified 25 PROMs for round 2. During this round, participants were asked to rank the remaining PROMs. Based on these rankings, the core research team finalized a list of 12 instruments planned for the study. In round 3, the final list was shared with participants along with the planned administration frequency and modality (Table 1). All Delphi participants agreed with the proposed schema in round 3. A comprehensive list of all PROMs considered all 3 rounds, citations, and additional literature can be found on the Delphi Kit website.

**Table 1:** The final list of patient-reported outcome measures selected from the e-Delphi process for patients and families with type 1 diabetes using continuous glucose monitors.

Domain and instrument									Items, n			Age (years)			Scheduled
**Diabetes distress**															
	**Problem Areas in Diabetes scale**														Annually, emergency department visit, and hospitalization
		Child						20			8-11				
		Youth						26			>12				
		Parent						26			Not restricted				
	Diabetes Distress Scale							17			>18				
	**Hypoglycemia Fear Survey**														Hospitalization
		Child						25			6-18				
		Parent						28			Not restricted				
	**Blood Glucose Monitoring Communication questionnaire**														Hospitalization and emergency department visit
			Child					8			8-18				
			Parent					8			Not restricted				
**Autonomy**															
	Diabetes Knowledge Test							19			12-18			Baseline and transitional milestones	
	The Mercy What I Know About Diabetes							23			>18				
**General health and quality of life**															
	**Type 1 Diabetes and Life measures**														Annually
						Child		21			8-11				
						Adolescent		23			12-17				
						Parent		22			—				
**Psychosocial**															
	Patient Health Questionnaire-9							9			>12			Annually	
	**Diabetes Family Responsibility Questionnaire**														Annually
					Child		17			8-18					
					Parent		17			Not restricted					
	**Diabetes Strengths and Resilience Measure**														Annually
					Child		12			9-12					
					Adolescent		12			13-17					
					Young adult		16			18-22					
**Technology acceptance**															
	**Diabetes Technology Attitude**														Annually
				Youth				5			>12				
				Parent				5			Not restricted				
	Glucose Monitoring Satisfaction Survey							15			>12			Baseline	

The PedsQL 3.2 Diabetes module [25] was selected for inclusion in round 1, but reviewers reported concerns with validity, reliability, and length; hence, it was replaced by the Type 1 Diabetes and Life (T1DAL) measures [26] during round 2 on the suggestion of one of the Delphi participants and was approved by the other participants in round 3. One of the Delphi participants was a subject matter expert on adolescents with diabetes and suggested during round 2 that the Problem Areas in Diabetes scale (PAID) was more geared toward the pediatric population and the Diabetes Distress Scale (DDS) is a more suitable instrument for adolescents. Therefore, we added the DDS for patients older than 18 years. The Diabetes Technology Attitude survey was selected over the FDA-recommended INSPIRE Questionnaires [11] because of the latter’s focus on AID systems. In total, 7 out of 12 instruments were selected to be administered annually. To minimize the burden on patients and parents and reduce survey fatigue, all instruments are staggered across quarters so that families complete no more than 3 surveys at a time. A brief description of each domain is presented here, and a detailed description of each of the 12 instruments is provided in Multimedia Appendix 1.

### Diabetes Distress and Burden Domain

T1D can cause significant distress for patients and their families, particularly as it relates to diabetes regimen–specific duties including continuous glucose monitoring and adherence to meal, physical activity, and treatment management plans [27]. Social distress, diabetes-related fears, coping lifestyle, and financial burden also play a role in the overall psychosocial burden of diabetes. This distress and burden can manifest in the form of anger, guilt, frustration, denial, loneliness, and fear of hypoglycemia [28]. Thus, it can negatively affect the functioning, QoL, and ultimately glycemic control, leading to further deterioration of mental and physical health [29]. The purpose of this domain is to identify the emotional and psychosocial needs of patients and caregivers, create opportunities to discuss them with providers, and engage appropriate support mechanisms. Our Delphi panel selected 4 instruments, including the PAID [28,30], DDS [31], Hypoglycemia Fear Survey [32], and Blood Glucose Monitoring Communication questionnaire [33] to assess the distress in patients with T1D. The PAID will be administered annually to children (aged 8-12 years), youths (aged 12-17 years), and their parents. DDS will be administered annually to patients older than 18 years of age. The Hypoglycemia Fear Survey and Blood Glucose Monitoring Communication questionnaire will be triggered with events such as diabetes-related hospitalization or emergency department visits when patients may experience increased anxiety related to monitoring. Details regarding age validation and administration are described in Table 1.

### Autonomy Domain

Effective diabetes self-management (DSM) can prevent or delay diabetes-related complications. Autonomous motivation is important in adopting and maintaining DSM practices that improve glycemic control [34,35]. DSM requires continual improvement of disease-related knowledge in patients as well as maintaining engagement, skills, and self-efficacy [36]. DSM was identified as the principal construct to assess for this domain. The Delphi panel chose the Diabetes Knowledge Test [37] for the 12-18–year age group and The Mercy What I Know About Diabetes [38] for patients older than 18 years. DSM behaviors and perceived autonomy can change over time and also depend on support from family and health care providers [34]. Therefore, the group decided to administer these instruments at baseline and transitional milestones.

### General Health and QoL Domain

T1D requires a daily execution of complex tasks owing to frequent glucose monitoring, insulin injection, dose adjustments, and carbohydrate estimation [39]. Long-term treatment management brings on physical and psychological hardship and impacts the QoL of individuals with T1D [40]. QoL-related PROMs in this domain assess developmentally appropriate emotional, physical, and social well-being and treatment satisfaction, and can help clinicians provide early intervention and health education, and prevent disease-related complications. The group chose to administer the T1DAL measures [26,41] annually to assess QoL in all age groups.

### Psychosocial Domain

Depression and anxiety are much more common in children with T1D, which negatively impact social life and well-being [42]. The American Diabetes Association has published evidence-based guidelines to help providers implement psychosocial assessments into the care of patients with diabetes and their families [43]. Patient-centered psychosocial care requires interactive communications, problem identification, psychosocial screening, diagnostic evaluation, and cognitive, behavioral, and social intervention to optimize health outcomes [43,44]. Positive and supportive parenting styles have been shown to improve QoL in patients with T1D [45]. This domain includes instruments to assess both risk and protective factors, as well as family dynamics. Select instruments include the Patient Health Questionnaire-9 for patients older than 12 years [46], the Diabetes Family Responsibility Questionnaire for children aged 8-18 years and their parents [47,48], and the Diabetes Strengths and Resilience Measure for all age groups [49-51]. All instruments in this domain will be administered annually at different time points.

### Technology Acceptance Domain

This is a critical but often neglected domain of T1D management. Medical devices including CGMs and insulin pumps are critical components of T1D management. However, patients often reported the barriers when using these devices on a daily basis. The most common barriers are related to the physical experience of these devices, including the hassle of wearing them, not wanting to wear them, and not liking how devices look on their bodies [52]. Given the potential of CGMs to improve glycemic control, it is important to assess and address barriers to device uptake. The Delphi panel selected the Glucose Monitoring Satisfaction Survey [53] to be administered at baseline and the Diabetes Technology Attitude [52] annually among all parents and children aged 12 years and older. Of note, we did not identify any CGM-specific instruments.

## Discussion

### Principal Findings

Pediatric patients with T1D and their families face a lifetime of lifestyle and behavior modifications, medical therapies, and complex treatment regimen to prevent T1D complications and mortality [54]. CGM can make self-management and correction simpler [2], but many patients feel distress related to the multitude of self-care responsibilities to optimize glycemic control, resulting in low self-efficacy and reduced self-care [55]. PROMs can give providers a structured method to evaluate the burden on patients, the impact of technology, and opportunities to identify patients who may benefit from additional support. The intersection of technology, patient behaviors, and PROMs has seen increased attention, with large national registries such as the BETTER Patient Engagement registry in Canada and the T1D Exchange registry in the United States, featuring these concepts prominently in their publications [56,57].

In 2017, the National Institute of Diabetes and Digestive and Kidney Diseases and the American Diabetes Association cosponsored a 2-day workshop to identify research priorities related to patient-reported outcomes for patients with diabetes and published their conclusions in 2019 [58]. The authors identified a number of themes relevant to pediatric diabetes, including shared disease management between parents and children, the transition to self-management as children age, the overall burden of disease with a focus on psychosocial impact, and the importance of including self-reported instruments, when possible, over parent proxy instruments. Another central theme identified in the paper was that it would be impossible to select a single approach to the development and selection of patient-reported outcomes for all uses; rather, it would be important to rely on contextual factors and to consider specific goals. We applied several of these themes to our own work, prioritizing self-reported outcomes over parent proxy when possible and ensuring that we included multiple options for assessing the psychosocial impact of T1D. The iterative comments from our Delphi participants also highlight the same point acknowledged by the workshop: there is no universal set of PROMs; rather, the specific goal and population should drive the selection of tools. Through this effort, because this study was undertaken as part of an FDA-funded real-world evidence demonstration project, we explicitly sought to identify a series of PROMs that could encompass the complex, holistic, and multifactorial perceptions of patients and families living with diabetes across a number of domains [59].

To our knowledge, this is the first attempt to leverage a systematic process to generate a list of PROMs specifically for pediatric patients with T1D using CGMs. In 2009, the Patient-Reported Outcome Measurement Group of the University of Oxford Department of Health published a structured review of PROMs for diabetes [10]. This review did not focus on pediatric patients or CGM users, but it identified 9 PROMs that could be used in pediatric patients with diabetes (4 generic and 5 diabetes-specific). While many of these instruments were included in round 1 in this study, none of them were present in our final list. This may be in part owing to the 10-year gap between the 2 projects and the development of new tools in the interim, such as the T1DAL. The National Institutes of Health also organized a group discussion of psychology experts to recommend a list of PROMs for T1D across all age groups for internal use (unpublished, personal correspondence). They organized 20 diabetes-specific and 15 “other relevant” instruments into 4 domains: diabetes distress and burden, psychosocial attitudes toward automated insulin delivery, hypoglycemia (worries, Fear, behavior, and confidence), and technology acceptance and satisfaction. Overall, 5 out of 12 of our final instruments were also featured on their list.

There were a number of strengths to our approach. This was an asynchronous and digital process where we involved a large variety of stakeholders from different fields. The decision process was transparent, technologically sophisticated, and very well documented. Of note, we conducted our asynchronous digital process prior to the COVID-19 pandemic, which, in many ways, prepared us for some of the unique workflow adaptations that we undertook after March 2020. All of the final instruments included in this study have been used in research settings, some having been used quite extensively. Ultimately, we prioritized instruments that were relatively short, easy to administer electronically, and those that address tangible clinical concepts so that the battery of surveys could be useful to clinicians and researchers focused on clinical, translational, and implementation research. Multimedia Appendix 1 provides descriptions of each instrument and a summary of the underlying evidence to support clinicians and researchers interested in implementing PROMs in their practice. One strength of the Delphi process is that the responses are weighted equally, providing controlled feedback on group opinions and reducing subjective bias.

### Limitations

There were also several limitations to our study. The Delphi process can be quite time-consuming and laborious for participants. Participant attrition in our own study was quite high; only 1 in 5 participants made it to the final round. The final 4 participants were 2 pediatric endocrinologists and 2 pediatric psychologists specializing in the care of children and adolescents with T1D. This group was reasonably representative of the initial panel of participants, although notably lacking dieticians and industry representatives. Though we did not formally collect data on why participants did not complete the process, anecdotally, many of them cited that the project was time-consuming. In the future, it would be important to address engagement and retention through decreased time burden and increased engagement and compensation. Many participants reported not being familiar with the included instruments prior to the Delphi process; however, given their expertise in the field, we believe that their recommendations are still valid and helpful. Our study was specifically focused on pediatric patients with T1D using CGMs; hence, our findings may not be generalizable to other populations, such as adults with T1D or children with type 2 diabetes or to explore outcomes for patients with insulin pumps or automated insulin dosing systems. Lastly, identifying PROMs is only the beginning of this process; patients and providers will need training and education on the importance and role of PROMs and how to administer and complete the instruments, interpret the instruments, and incorporate them into interactional decision-making with patients and families. It should be noted that patients or patient representatives did not participate in this Delphi process, although patients were involved in the development of many of the instruments selected for this study. This decision was made on the basis of the need for Delphi participants to have extensive subject matter expertise in research methods and experience with reading and reviewing of medical literature to evaluate the PROMs. Our initial goal was to work with clinical and research experts to develop a curated library of PROMs that future studies could select from. These studies would then involve patients or a community advisory board in selecting the PROMs from the curated library, which are most appropriate for the population and the study aims. Our implementation strategy includes the administration of these surveys at home, in the clinic, and shortly after hospitalization or emergency department visits using REDCap and obtaining feedback from the patients. We plan to administer all PROMs electronically along an adaptive schedule to minimize patient burden and refusal, similar to Corathers et al [4]. We have also developed capabilities to display REDCap PROM data in HealtheIntent alongside clinical data. The interactional dashboard that includes all of these sources will enable the visualization and analysis of individual patients as well as cohort-level data from medical records, devices, and patients.

### Conclusions

PROMs can provide critical insights into the psychosocial well-being of patients, and their role in both clinical care and research is becoming more important. National registries, federal agencies, and philanthropic organizations have all placed increased focus on the use of PROMs to measure care quality and patient engagement in their care. This study is the first to provide guidance and resources for clinicians and researchers on selecting PROMs that are specific for CGM use among pediatric patients with T1D. Future studies will need to focus on refining and expanding this battery of instruments with additional concepts. The selection of specific PROMs from this list should be made in collaboration with patients and patient representatives to ensure that they are fit for purpose and appropriate for the population of interest.

## Appendix

Multimedia Appendix 1 A brief description of the final patient-reported outcome measures selected from the e-Delphi process for patients and families with type 1 diabetes using continuous glucose monitors.

## Abbreviations

AID: automated insulin dosing
CGM: continuous glucose monitor
DDS: Diabetes Distress Scale
DSM: diabetes self-management
e-Delphi: electronic Delphi
EHR: electronic health record
FDA: Food and Drug Administration
INSPIRE: Insulin Dosing Systems: Perceptions, Ideas, Reflections, and Expectations
PAID: Problem Areas in Diabetes scale
PROM: patient-reported outcome measure
QoL: quality of life
T1D: type 1 diabetes
T1DAL: Type 1 Diabetes and Life
