# Supplementary material for: Modified e-Delphi Process for the Selection of Patient-Reported Outcome Measures for Children and Families With Type 1 Diabetes Using Continuous Glucose Monitors: Delphi Study
Source: JMIR Diabetes. 2022 Nov 30;7(4):e38660. doi: 10.2196/38660 (PMC9752458; doi:10.2196/38660)
Supplement: Multimedia Appendix 1 [file diabetes_v7i4e38660_app1.docx]

**Supplemental File:**

**APPENDIX A**

A brief description of the final patient-reported outcome measures selected from the e-Delphi process for patients and families with type 1 diabetes using continuous glucose monitors.

**Diabetes distress domain:**

**1. Problem Areas in Diabetes (PAID)-**This is a validated and reliable (Cronbach’s a ≥ 0.93 for the total scale) 20-item self-reported instrument for measuring diabetes-related emotional distress and covers a range of negative emotional problems of patients with diabetes[28]. It also shows a stronger focus on food-related problems and complications. PAID is being administered to parents to examine their diabetes burden [30]. Respondents rate the severity of each problem on a five-point Likert scale (0, ‘not a problem’ to 4, ‘serious problem’; higher scores indicate more distress.

**2. Diabetes Distress Scale (DDS)-** The DDS is more reflective of physician-related distress and problems concerning diabetes self-management [31]. Moreover, it has a stronger focus on motivational and behavioral problems associated with diabetes self-management, and stronger associations with self-care activities and metabolic outcomes. It is a reliable and validated (eg. Cronbach’s a ≥ 0.88) 17-item with 5-point Likert scale same as PAID, indicating higher score is higher distress. For the same reasons, we decided to administer this instrument >18 years of age where self-management and self-care are important aspects of during and after transition.

**3. Hypoglycemia Fear Survey (HFS) -** This instrument is being used to assess the level and impact of fear in children (CHFS) with diabetes as well as their parents (PHFS). The nocturnal hypoglycemia episodes in children can negatively impact quality of life, emotional wellbeing, and neurocognitive functioning of a child. The episodes of severe hypoglycemia have been reported during school hours when the child is supervised by the parent or adult with limited knowledge about diabetes management creates a fear in parents as well [32]. This may result in increased anxiety about diabetes management, obsessive self-monitoring, deliberately keeping BG levels too high, dependence on others, feelings of guilt and frustration, a sense of loss of control, embarrassment, relationship stress and avoidant behavior. The purpose of having this instrument was to administer EMA after an event such as hospitalization, and not annually because the anxiety/ fear is usually higher after such events and patients/parents may require behavioral intervention and education to reduce occurrence. The total CHFS and PHFS reliability scores are relatively higher in all age groups (e.g. Cronbach’s a ≥ 0.84) [32]. These instruments also provide two subscales- the Behavior subscale (HFS-B), which measures behaviors used to avoid hypoglycemia and its negative consequences and the Worry subscale (HFS-W), which measures different anxiety-provoking aspects of hypoglycemia.

**4. Blood Glucose Monitoring Communication (BGMC) Questionnaire-** This is reliable, valid, and stable 8-items questionnaire (Cronbach’s a for youth =0.77 and parents =0.82) with corresponding responses on a three-point Likert scale (1 = almost never, 2 = sometimes, 3 = almost always) [33]. Total scores can range from a minimum of 8 (indicating no negative affect) to 24 (indicating a high level of negative affect). The instrument assesses the affective responses to BGM results experienced by youths and their parents. Parents and patients are being asked to report their negative feelings when they experienced high blood sugar in the past week. Therefore, we are planning to administer them as an EMA after patients get hospitalized or ED visit.

**Autonomy domain:**

**5. Diabetes knowledge test (DKT-2)**-Michigan-DKT-2 is a 23-item reliable and valid instrument to assess the patient’s or populations’ general knowledge about diabetes and self-care [37]. It has two subscales- 1-14 item general test (alpha=0.77) and 15-23 item insulin use (alpha=0.84). The test asks patients to choose one answer from multiple choice questions. It is a quick and low-cost instrument to assess the diabetes knowledge in adult patients with type-1 diabetes.

**6. The Mercy What I Know About Diabetes (M-WIKAD)-** M-WIKAD is a new and valid (Cronbach’s alpha = 0.70) 19-item questionnaire for adolescents focusing on advanced problem-solving, hypoglycemia prevention and management, taking insulin/medication administration, daily management and healthy active living variables [38]. We will administer this for patients with Diabetes of 12-18 years of age. The questions assess the patient's knowledge –eg. “When should you check your blood glucose?’, “If your blood glucose is below 70, what should you do?”

**General Health and Quality of Life (QOL) domain:**

**7. Type 1 Diabetes and Life (T1DAL) Measures-** T1DAL is a new, valid (Cochran’s α = 0.84 and 0.89 for children and adolescents respectively) instrument to use in research and clinical care [26,41]. It measures positive and negative constructs, including diabetes strengths and burden. The instrument asked the questions on 5-point Likert scale 1= No, not at all true to 5= Yes, very true. The questions are focused on diabetes and peer relationship, sources of support, financial consideration, emotional experience, and daily activities. The total score can be calculated on a scale of 0 to 100 and subscales are also calculated on a scale of 0-100. The instrument will be administered for both patients and parents.

**Psychosocial domain:**

**8. Patient Health Questionnaire-9 (PHQ-9)-**This is a 9-item valid, reliable (Cochran’s alpha =0.89) instrument for assessment of mental disorders [46]. The score can range from “0” (not at all) to “3” (nearly every day) and scores of 5, 10, 15, and 20 represented mild, moderate, moderately severe, and severe depression, respectively. Clinical guidelines advise screening for depression in patients with diabetes therefore PHQ-9 is widely being used in clinical settings.

**9. Diabetes Family Responsibility Questionnaire (DFRQ)-**This is a valid 17-item instrument (total scale Cochran’s alpha =0.85) to examine how youth with type 1 diabetes and their caregivers share responsibilities around diabetes management [47,48]. The three factors included responsibilities related to regimen tasks, General Health Maintenance (direct management tasks), and Social Presentation (indirect management task) of diabetes. Both caregivers and patients complete equivalent forms and assign one of the following values: 1 = child taking or initiating responsibility for this task almost all the time; 2 = caregiver and child sharing responsibility for this task almost equally; 3 = caregiver taking or initiating responsibility for this task almost all the time. Scores can range from 17 (adolescent has complete responsibility) to 51 (caregiver has complete responsibility). A score of 34 indicates equal sharing of responsibilities.

**10. Diabetes Strengths and Resilience (DSTAR)-**This is a 12-item simple, reliable, and consistent (alpha =0.89 for total some score) instrument assessing diabetes-related strengths, including confidence in one’s own abilities and access to help from close others [49]. Responses are based on “the answer that tells about you best” using a 5-point scale (1 = never to 5 = almost always). The total score and sub score can range from 12-60. This instrument will be administered to children [49], teens [50] as well as young adults [51] Higher scores on specific points indicate that the patient has greater strength to cope up when diabetes related challenges arise.

**Technology acceptance domain:**

**11. Diabetes Technology attitude (DTA)** – It is assessed with five items where each item was rated on a 5-point Likert scale to indicate agreement with the statement. Example items are as follows: “Diabetes technology has made my life easier,” “I am lucky to live in a time with so much diabetes technology.” Higher scores indicated more positive attitudes about devices and technology. Internal consistency for this instrument was 0.93 for general technology attitudes and 0.91 for diabetes-specific technology attitudes [52]. Since all the participants will be assessed for technology use and ease at study recruitment, we will administer this survey annually. We have planned to administer this instrument in the teenage population and adults as well as parents will respond on behalf of minors.

**12. Glucose Monitoring Satisfaction Survey (GMSS)** - GMSS is a 15-scale valid and reliable instrument (Total Cronbach’s a = 0.86 and subscales Cronbach’s a > 0.86) to examine the psychological impact and perceived satisfaction using glucose devices and to determine the acceptability of such devices and its impact on quality of life [53]. The GMSS comprises four clinically meaningful subscales that capture key features contributing to device satisfaction- openness (4-items), emotional burden (4-items), behavioral burden (4-items) and trust (3-items). It is 5-point Likert scales varying from strongly disagree to strongly agree. The higher score indicates greater well-being, lower level of diabetes distress and greater satisfaction towards BG monitoring. We will administer this instrument at baseline only to assess the patient's attitude and perception towards CGM.
